# Fracture Resistance of Zirconia-Reinforced Lithium Silicate in Single Posterior Implant-Supported Crowns: An In Vitro Study

**DOI:** 10.3390/dj13120574

**Published:** 2025-12-03

**Authors:** Maria Dolores Gómez-Adrián, Pasquale Natale, Alberto Molina-Palomero, Ángel Vicente-Escuder, Julián Espinosa-Giménez, Blanca Gil-Marqués, Marcelino Pérez-Bermejo, Carolina Larrazabal-Morón, Javier Barberá-Millán, Lucía Miralles-Jordá

**Affiliations:** 1 Oral Surgery Unit, Dentistry Departament, Faculty of Medicine and Health Sciences, Catholic University of Valencia San Vicente Mártir, C/Quevedo No. 2, 46001 Valencia, Spain; mariadolores.gomez@ucv.es (M.D.G.-A.); julian.espinosa@ucv.es (J.E.-G.); blanca.gil@ucv.es (B.G.-M.); carolina.larrazabal@ucv.es (C.L.-M.); javier.barbera@ucv.es (J.B.-M.); lucia.miralles@ucv.es (L.M.-J.); 2 Oral Surgery Master, Dentistry Departament, Faculty of Medicine and Health Sciences, Catholic University of Valencia San Vicente Mártir, C/Quevedo No. 2, 46001 Valencia, Spain; pasquale.natale@mail.ucv.es; 3 Oral Surgery Unit, Dentistry Departament, Faculty of Medicine and Dentistry, University of Valencia, Blasco Ibañez Avenue No 15, 46010 Valencia, Spain; alberto.molina@fundacions.uv.es; 4 Instituto de Tecnología de Materiales, Universidad Politécnica de Valencia, 46022 Valencia, Spain; avicente@mcm.upv.es; 5 SONEV Research Group, Faculty of Medicine and Health Sciences, Catholic University of Valencia San Vicente Mártir, C/Quevedo No. 2, 46001 Valencia, Spain

**Keywords:** zirconia, fracture resistance, CAD/CAM, implant-supported restorations, dental ceramics

## Abstract

**Background/Objectives:** Zirconia-reinforced lithium silicate (ZLS) is a glass–ceramic that combines the translucency of lithium disilicate with the enhanced strength provided by dispersed zirconia crystals. Evidence on the mechanical behavior of ZLS implant-supported crowns remains limited. This study evaluated the fracture resistance of posterior monolithic ZLS crowns and analyzed the influence of cement type and artificial aging. **Methods:** Forty ZLS crowns (Celtra™ Duo) were fabricated, cemented onto straight titanium abutments and assigned to four groups (*n* = 10) according to cement type (adhesive or self-adhesive) and aging (500,000 cycles at 150 N). Specimens underwent axial load-to-fracture testing using a universal testing machine. Data were analyzed with Student’s *t*-tests and Kruskal–Wallis tests (α = 0.05). **Results:** Mean fracture resistance was 1625.46 ± 340.02 N. Although adhesive cement showed higher mean values than self-adhesive cement, no statistically significant differences were found (*p* = 0.102). Artificial aging also produced no significant reduction in fracture resistance (*p* = 0.674). All groups exceeded the physiological posterior occlusal forces. **Conclusions:** Monolithic ZLS crowns cemented onto titanium abutments demonstrated high fracture resistance under axial loading. Within the limitations of this in vitro design, neither cement type nor mechanical aging significantly affected performance. These findings support the mechanical feasibility of ZLS for posterior implant-supported single crowns, although further studies including thermomechanical aging and oblique loading are required.

## 1. Introduction

Metal–ceramic crowns have traditionally been the standard for implant-supported prostheses due to their strength and long-term predictability [1,2,3]. However, the growing demand for highly esthetic and metal-free restorations has accelerated the development of advanced monolithic ceramics with improved optical and mechanical properties.

In recent years, monolithic CAD/CAM materials such as lithium disilicate (LD) and zirconia-reinforced lithium silicate (ZLS) have gained relevance for implant-supported crowns because they avoid the veneer chipping commonly associated with bilayer ceramics while offering improved translucency and adequate mechanical strength.

Chipping of the veneering ceramic remains the main technical complication of all-ceramic restorations. CAD/CAM (computer-aided design/computer-aided manufacturing) technology enables the fabrication of monolithic restorations that eliminate veneering layers, reduce internal defects, and produce more homogeneous structures, which improves resistance to fracture and clinical longevity [4]. Zirconia-reinforced lithium silicate (ZLS) is an emerging glass–ceramic that consists of a lithium silicate glass matrix containing uniformly dispersed zirconia nanocrystals (8–10%). These tetragonal zirconia particles act as local reinforcements that deflect crack propagation pathways, increasing fracture toughness, while their submicrometric size preserves translucency by minimizing light scattering. This hybrid microstructure gives ZLS an intermediate combination of translucency and mechanical strength between lithium disilicate and monolithic zirconia.

This hybrid microstructure yields flexural strength values of approximately 210–370 MPa and an elastic modulus around 70 GPa, positioning ZLS between lithium disilicate and zirconia in terms of optical and mechanical behavior [5,6,7,8].

Although numerous studies have evaluated lithium disilicate or zirconia on implants, evidence specific to monolithic ZLS posterior implant crowns remains limited. Despite the increasing use of ZLS, few studies specifically evaluate its fracture behavior in posterior implant-supported single crowns. In addition, the available in vitro data are heterogeneous in terms of abutment design, cementation protocols and fatigue methods, making direct comparison difficult. This gap highlights the need for controlled investigations that isolate the influence of cement type and mechanical aging on the fracture behavior of monolithic ZLS crowns. The literature on ZLS implant-supported restorations is still sparse, and the available in vitro studies show considerable variability in methodology, abutment type, cementation protocols, and fatigue conditions [9].

The present study focused on the influence of cement type and artificial aging. Cement type may affect fracture resistance by modifying the elastic modulus and stress distribution at the crown–abutment interface, while cyclic loading allows simulation of mastication-related fatigue. By standardizing other clinically relevant variables (crown morphology, minimum thickness, abutment design, load direction, antagonist material), these two factors can be isolated for controlled comparison [10].

Because ZLS exhibits a reinforced microstructure with zirconia nanocrystals that enhance crack resistance and distribute stresses more uniformly, we hypothesized that variations in cement type or moderate cyclic aging would have limited influence on fracture resistance when restorations are fabricated with adequate thickness and supported by rigid titanium abutments. Therefore, the null hypothesis stated that neither cement type (adhesive vs. self-adhesive) nor mechanical aging would significantly affect the fracture resistance of ZLS posterior implant-supported crowns.

The objective of this study was therefore to evaluate the fracture resistance of monolithic ZLS single posterior implant-supported crowns and to determine the influence of cement type and artificial aging on their mechanical behavior.

## 2. Materials and Methods

### 2.1. Study Design

An in vitro experimental study was conducted to evaluate the fracture resistance of zirconia-reinforced lithium silicate (ZLS) implant-supported posterior single crowns, taking into account the type of cement used and the effects of artificial aging. A total of 40 samples were prepared and equally distributed into four experimental groups (*n* = 10) according to the studied variables.

The sample size (*n* = 10 per group) was based on previous in vitro studies evaluating fracture resistance of ZLS or lithium silicate-based implant crowns, which commonly use sample sizes between 6 and 12 specimens per group to obtain adequate statistical power in monotonic load-to-fracture tests. This number has been shown to provide stable variance estimates for ceramic materials tested under standardized conditions. Although no formal power analysis was conducted, the selected sample size follows established methodological standards in the literature and is consistent with comparable investigations.

### 2.2. Sample Preparation

**Figure 1:**
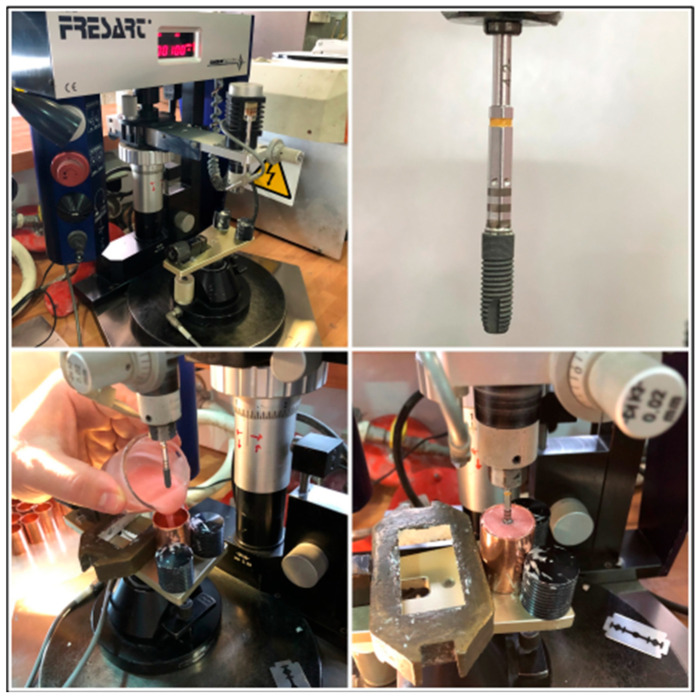
From (**top**) to (**bottom**) and (**left**) to (**right**). Parallelizer; implant with conveyor; pouring of the light-curing resin; setting of the resin around the implant in the correct position.

Each sample consisted of an internally connected titanium dental implant (4.2 mm diameter) fixed in a copper metal cylinder. The cylinder was filled with a self-curing polymethyl methacrylate (PMMA) resin, commonly used in in vitro implant studies due to its dimensional stability, although its elastic modulus is lower than that of human cortical bone. All implants were positioned vertically and parallel using a parallelizer to ensure uniform orientation with respect to a central reference plane. This procedure ensured the correct alignment of the crowns during subsequent tests (Figure 1).

In accordance with the manufacturer’s recommendations, acrylic resin was poured before inserting each implant to ensure adequate adhesion and stability within the metal cylinder. In accordance with the manufacturer’s instructions, a straight titanium abutment (6 mm in diameter and 2 mm in transgingival height) was screwed onto each implant with a torque of 25 Ncm using a torque wrench to ensure stable fixation.

### 2.3. Design and Manufacture of the Crowns

**Figure 2:**
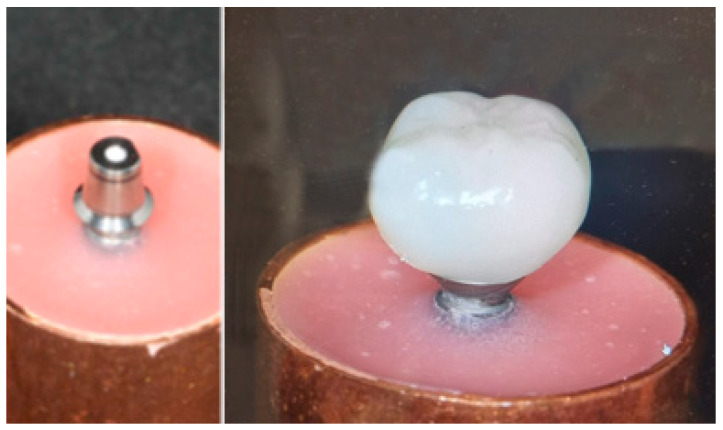
Final specimen configuration used for mechanical testing. (**Left**) titanium abutment fixed in the acrylic resin block. (**Right**) monolithic ZLS crown cemented onto the straight titanium abutment after the cementation protocol. All specimens were fabricated with standardized crown morphology and identical abutment geometry.

A total of 40 crowns with mandibular first molar morphology were designed following standardized dimensional values: Cervico-occlusal length: 7.5 mm; mesiodistal diameter: 11 mm; and vestibulo-lingual diameter: 10.5 mm. These dimensions were selected to reproduce the anatomical characteristics and ensure a proper fit on the abutments (Figure 2).

The design was created by optically scanning the abutment with the CEREC^®^ Omnicam and digitally processing it with CEREC^®^ Software 4.6. Following the material manufacturer’s recommendations to prevent fractures due to insufficient thickness, a minimum thickness of 1.5 mm was ensured in the axial walls and cusps.

The crowns were milled using Celtra™ Duo blocks (Dentsply Sirona, Charlotte, NC, USA) in the CEREC^®^ MC XL system (Dentsply Sirona, USA). The chemical composition of the material was: SiO_2_ (56.1–61.8%), P_2_O_5_ (5.3–6.5%), Al_2_O_3_ (1.7–2.2%), Li_2_O (16.2–20.9%), K_2_O (1.7–2.2%), ZrO_2_ (9.3–10.5%), CeO_2_ (0–1.9%), and Tb_4_O_7_ (0.9–2.1%). The material has a flexural strength between 210 and 370 MPa and an elastic modulus of 70,000 MPa.

### 2.4. Experimental Groups

The 40 samples were divided into four groups (*n* = 10) according to the type of cement used and whether artificial aging was applied.

- CUE: Crowns made with Celtra™ Duo and cemented with Calibra^®^ Universal (Dentsply Sirona Iberia S.A., Barcelona, Spain) were subjected to artificial aging.
- CUNE: Celtra™ Duo crowns were cemented with Calibra^®^ Universal (Dentsply Sirona Iberia S.A., Barcelona, Spain) and were not artificially aged.
- CCE: Celtra™ Duo crowns that were cemented with Calibra^®^ Ceram (Dentsply Sirona Iberia S.A., Barcelona, Spain) and were subjected to artificial aging.
- CCNE: Celtra™ Duo crowns cemented with Calibra^®^ Ceram (Dentsply Sirona Iberia S.A., Barcelona, Spain) without artificial aging.

### 2.5. Surface Preparation and Cementation Procedure

Before cementation, the abutment surfaces were sandblasted with 50 µm aluminum oxide particles using a clinical unit at a pressure of 1 bar from a distance of 10 mm. A Teflon ball was used to protect the abutment from damage during sandblasting. After sandblasting, the samples were ultrasonically cleaned in distilled water for five minutes and dried with compressed air.

The inner surfaces of the crowns were etched with 5% hydrofluoric acid for 60 s. Then, they were rinsed with pressurized water and dried for 20 s.

For the Calibra^®^ Ceram group, adhesive was applied to the inner surface of the crown and the abutment for 20 s. For the Calibra^®^ Universal group, no adhesive was applied. Then, resin cement was applied with self-mixing tips. Any excess was removed before light curing.

The selection of two resin-based cements with different bonding mechanisms was intentional in order to represent commonly used clinical strategies. The adhesive resin cement (Calibra^®^ Ceram) requires surface conditioning and bonding application, providing micromechanical and chemical adhesion to the ceramic. In contrast, the self-adhesive resin cement (Calibra^®^ Universal) simplifies the protocol by eliminating the need for an adhesive layer and relies on its intrinsic chemistry for bonding. These cements therefore represent two contrasting adhesive approaches typically used in clinical practice, allowing assessment of whether the bonding strategy influences the load-to-fracture performance of ZLS crowns.

Polymerization was performed with an LED lamp for 20 s on each side of the sample. In accordance with the recommendations of ADA Specification No. 96 (2000) and ISO 9917 [11], cementation was performed at 24 °C under an axial load of 5 kg for 10 min to ensure the quality of the bond.

The cemented samples were then stored in an incubator at 37 °C and 100% humidity for 24 h to complete the polymerization reaction and cement stabilization.

### 2.6. Cyclic Loading (Artificial Aging)

The cyclic loading test was performed at the Materials Technology Institute of the Polytechnic University of Valencia to simulate the functional fatigue associated with chewing. The test was conducted using an Instron^®^ 8874 servo-hydraulic axial-torsional fatigue machine (Instron Corp., Canton, MA, USA), which is capable of applying variable and cyclic loads in different directions.

Samples from the aged groups (CUE and CCE) underwent 500,000 cycles (equivalent to approximately two years of use in the mouth), applying a load of 150 N at a frequency of 5 Hz. The load was applied to the occlusal surface using a metal device designed in the laboratory to fit the occlusal morphology of the crowns.

Articulating paper was used to ensure uniform load distribution over the occlusal surface and simulate actual chewing conditions.

### 2.7. Fracture Resistance Test

After artificial aging, all samples underwent a vertical compressive load test until fracture. This test was performed using a Shimadzu^®^ AG-100 kN universal testing machine (Shimadzu Corp., Kyoto, Japan) equipped with a 100 kN load cell. The load cell was connected to a computer running TRAPEZIUM-X^®^ version 1.00 software (serial number 942356CA).

The samples were secured with a screw-type anchoring system to ensure their immobility during the test. The axial load was gradually applied at a displacement rate of 0.5 mm/min until the restoration fractured. The load values (in N) and load-deformation curves were recorded during the process, which allowed us to identify the exact fracture point and analyze the mechanical behavior of each sample.

### 2.8. Statistical Analysis

A statistical analysis was performed using SPSS version 23 (IBM Corp., Armonk, NY, USA) at a 95% confidence level. Results with *p* < 0.05 were considered statistically significant.

The main dependent variable was fracture resistance in Newtons. Results are presented as means ± standard deviation (SD), expressed in newtons (N). The independent variables were the type of cement (resin adhesive or self-adhesive) and whether artificial aging was applied (yes or no).

Normality was assessed using the Shapiro–Wilk test, and homogeneity of variances was assessed using the Levene test. A *t*-test for independent samples was used to compare fracture resistance according to cement type and artificial aging application. When the normality assumption was not met, the nonparametric Kruskal–Wallis test was applied.

Given the relatively small sample size in each group (*n* = 10), special attention was paid to the assumptions of normality. Although the Shapiro–Wilk test indicated that the data were normally distributed, the interpretation of these results was made cautiously, as normality tests have limited power with small samples. Therefore, non-parametric analyses (Kruskal–Wallis test) were used whenever normality could not be confidently assumed, ensuring robustness of the statistical interpretation.

## 3. Results

A total of 40 zirconia-reinforced lithium silicate crowns (Cel-Tra™ Duo), which were cemented onto titanium abutments, were analyzed. The crowns were divided into four experimental groups according to the type of cement used (Calibra^®^ Ceram or Calibra^®^ Universal) and whether artificial aging was applied.

### 3.1. Overall Fracture Resistance

The mean fracture resistance of all crowns was 1625.46 ± 340.02 N, with values ranging from 968.63 N to 2368.43 N. These results are above the physiological range of masticatory forces in the posterior region, suggesting adequate resistance for clinical function.

### 3.2. Influence of Cement Type

Table 1 shows that although numerical differences were observed between groups, these did not reach statistical significance (*p* = 0.102). Therefore, no effect of cement type on fracture resistance can be established.

### 3.3. Influence of Artificial Aging

Table 2 shows that artificial aging did not significantly affect the fracture resistance of ZLS, indicating the material’s structural stability after two years of simulated masticatory function.

### 3.4. Combined Analysis (Cement Type + Aging)

Table 3 shows that although aged Calibra^®^ Ceram crowns exhibited the highest values (1770 N), variability between groups prevented statistical significance.

As the tables above show all groups showed mean fracture loads above 1500 N; however, individual values ranged from approximately 970 N to over 2000 N. This distinction between mean values and absolute minimums clarifies the variability observed within each group. Even the lowest individual values remained within or near the upper limits of reported posterior masticatory forces, supporting the potential clinical relevance of the results.

This range is considered adequate for posterior implant-supported crowns. These results confirm the mechanical viability of ZLS for restorations with high occlusal loads, even after artificial aging.

**Figure 3:**
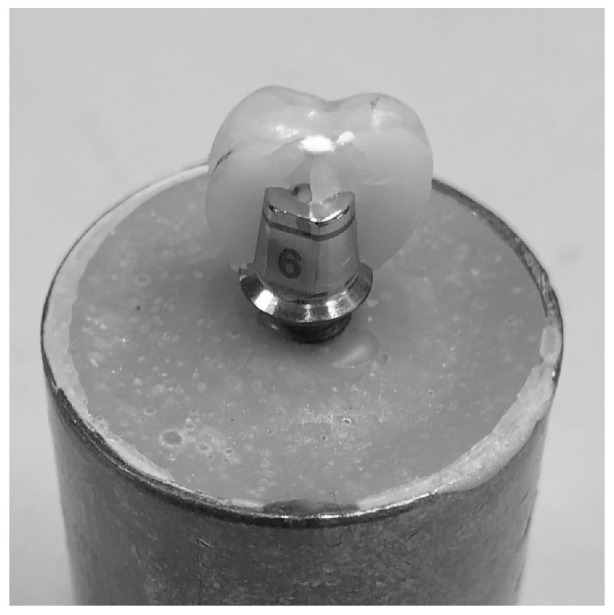
Representative macroscopic view of a cohesive fracture pattern in a monolithic ZLS crown after load-to-fracture testing. The crack origin can be observed at the occlusal loading point, propagating toward the axial walls without evidence of adhesive failure or abutment deformation.

All fractures were cohesive within the ceramic material. No debonding of the cement layer or deformation of the titanium abutment was observed in any specimen. Fracture lines typically originated at the occlusal loading point and propagated toward the axial walls, exhibiting the characteristic radial crack pattern described in monolithic glass–ceramic crowns (Figure 3).

## 4. Discussion

### 4.1. Interpretation of the Results

The present study evaluated the effect of cement type and artificial aging on the fracture resistance of monolithic ZLS implant-supported crowns. All groups exhibited mean fracture loads above 1500 N, while individual values ranged from approximately 970 N to 2360 N. These values remain above or within the upper limit of posterior occlusal forces reported in adults (500–900 N) [12].

The absence of significant differences between adhesive and self-adhesive cement groups suggests that, under the conditions of this study, the cement layer played a limited role in load-to-fracture behavior. This may be attributed to the monolithic ZLS structure and the rigid titanium abutment, which dominate the stress distribution patterns.

Although aging did not reduce fracture resistance, this result should be interpreted cautiously. The lack of an aging effect likely reflects the moderate magnitude of cyclic loading and the absence of thermal fluctuations, rather than an inherent insensitivity of ZLS to fatigue processes [13].

Overall, these findings indicate that single posterior ZLS crowns cemented onto titanium abutments can resist forces that exceed normal functional loading, regardless of cement type or mechanical cycling within the parameters used.

The exclusively cohesive failure mode observed suggests that fracture resistance was primarily governed by the intrinsic properties of the ceramic material rather than the cement interface. This may explain the absence of significant differences between cement types.

### 4.2. Comparison with Previous Literature

The fracture resistance values obtained in this study are consistent with previous in vitro investigations evaluating ZLS or similar glass–ceramics on implant abutments. Preis et al. reported mean failure loads for ZLS-based crowns exceeding 1500 N under cyclic loading conditions [14]. Gomes et al. also found that monolithic ZLS implant-supported crowns demonstrated favorable misfit behavior and high fracture loads, supporting their suitability for posterior single-unit restorations [15].

Previous work suggests that lithium disilicate typically exhibits flexural strength around 350 ± 50 MPa, whereas ZLS ranges between 210 and 370 MPa [2].

Therefore, ZLS cannot be described as mechanically superior to lithium disilicate. Instead, its performance is best characterized as intermediate, offering a balance between translucency and strength that is advantageous in posterior monolithic restorations.

Nonetheless, variation among studies is substantial due to differences in crown geometry, abutment material, cementation, and aging protocols, which complicates direct comparison across investigations.

### 4.3. Influence of Cement Type

The type of cement did not significantly affect the fracture resistance of the ZLS crowns. Although the mean fracture load was slightly higher for the adhesive cement group, the difference was not statistically significant. This absence of effect aligns with recent findings indicating that, in monolithic restorations with sufficient thickness, the intrinsic mechanical properties of the ceramic material play a more dominant role than the cementation protocol [16].

This result may be attributed to several factors, including the geometric homogeneity of the crowns, the purely axial loading applied in this study, and the uniform internal adaptation achieved via CAD/CAM fabrication. Under these conditions, differences in adhesive strategy may exert a limited influence on stress distribution.

Furthermore, ZLS exhibits an intermediate elastic modulus between lithium disilicate and zirconia, which may reduce the sensitivity of the crown–cement interface to variations in bonding strategy [17].

Under purely axial loading, stresses are largely compressive and transmitted directly through the ceramic material and the titanium abutment. These conditions minimize shear stresses at the cement interface, which likely contributed to the lack of difference between adhesive and self-adhesive cements.

In clinical situations, however, where loads are multidirectional and crown thickness or cement space may vary, the effect of cement type could be more relevant. Therefore, future studies should evaluate different geometric configurations, oblique loading, and variation in cement thickness to determine whether adhesive protocols may influence failure modes in more demanding clinical scenarios [18].

### 4.4. Mechanical Behavior of ZLS

ZLS combines a glass matrix with lithium silicate crystals reinforced by dispersed zirconia particles. This hybrid microstructure influences its mechanical performance and distinguishes it from both lithium disilicate and polycrystalline zirconia.

Recent microstructural analyses indicate that the zirconia phase contributes to modest crack deflection, although its toughening effect remains significantly lower than that of fully stabilized zirconia ceramics [19].

The reported flexural strength of ZLS (approximately 210–420 MPa) is within the range required for posterior monolithic crowns. Contemporary data show that lithium disilicate demonstrates flexural strength values around 350–450 MPa, whereas ZLS typically ranges from 210 to 420 MPa [20]. Therefore, ZLS cannot be described as mechanically superior; rather, it achieves a clinically useful balance between translucency and strength.

The presence of dispersed zirconia nanocrystals may delay crack propagation by inducing local crack deflection, although this mechanism is less pronounced than the transformation toughening observed in fully stabilized zirconia.

When comparing the present findings with previous in vitro studies, the fracture resistance of monolithic ZLS crowns is generally higher than that reported for conventional lithium disilicate, which typically ranges between 1000 and 1500 N in posterior implant-supported designs with similar thicknesses. This difference is consistent with the additional crack-deflection capacity provided by the dispersed zirconia nanocrystals in the ZLS matrix. Nevertheless, the values obtained in this study remain below those described for monolithic translucent zirconia, which frequently exceed 2000 N under comparable loading conditions due to zirconia’s higher fracture toughness and transformation-toughening behavior. Therefore, ZLS can be positioned as an intermediate material: stronger than lithium disilicate, with mechanical behavior approaching the lower range of zirconia-based ceramics, but still maintaining superior translucency compared with polycrystalline zirconia. This relative positioning aligns with the hybrid nature of its microstructure and supports its suitability for posterior implant-supported crowns in patients without high parafunctional loads.

The lower elastic modulus of ZLS (approximately 65–70 GPa) compared with zirconia provides a more elastic response under stress, potentially reducing stress concentration at the abutment–crown interface. This characteristic may partially explain the absence of catastrophic failures in the present study following mechanical aging [19].

However, the interpretation of the results should be cautious. Fatigue studies indicate that glass–ceramics—ZLS included—remain susceptible to subcritical crack growth, especially under combined thermal and mechanical loading conditions that were not assessed in the present protocol [21].

Taken together, these findings support the classification of ZLS as a material that balances esthetic performance and acceptable mechanical behavior for posterior implant-supported crowns, provided that appropriate thickness and occlusal design are maintained.

### 4.5. Limitations of the Study

The findings of this study must be interpreted within the constraints of its in vitro design. First, the supporting structure was fabricated using polymethyl methacrylate (PMMA), a material with a substantially higher elastic modulus than human cortical bone. This discrepancy may have influenced stress distribution at the implant–abutment–crown assembly, particularly under high-load conditions [22].

Second, all specimens were loaded exclusively under axial forces. However, masticatory function involves complex multi-directional and oblique loading patterns that generate different stress trajectories and fracture behaviors. The absence of non-axial loading may therefore underestimate clinically relevant failure modes.

Third, the aging protocol consisted of mechanical cycling alone, without thermocycling. Temperature fluctuations and moisture exposure are known to accelerate subcritical crack growth in glass–ceramics, and their omission limits the ability of this study to simulate long-term oral degradation.

The crowns were designed with standardized thickness and internal adaptation parameters. This ensured homogeneity among samples but limited the evaluation of geometric variability. In clinical practice, crown morphology, cement-space distribution, occlusal relief, and antagonist characteristics vary substantially and may influence mechanical behavior.

Only two cementation strategies were evaluated. Although this allowed a controlled comparison, it does not reflect the full range of contemporary adhesive protocols used for monolithic restorations. Further studies should assess different resin cements, primer systems, and cement-space configurations.

Additionally, mechanical properties of zirconia-reinforced lithium silicate vary across commercial systems, as demonstrated in recent comparative analyses [23]. Therefore, the results of this study apply specifically to the ZLS material tested and should not be indiscriminately generalized to other glass–ceramics.

Finally, the fracture test employed monotonic loading until failure. This method accurately reflects the clinical fracture process. While useful for standardization, monotonic loading does not reproduce cumulative fatigue damage or crack propagation dynamics occurring under prolonged clinical function.

Taken together, these considerations underscore the need for cautious extrapolation of the present findings to clinical settings, particularly in patients with parafunctional habits or high occlusal demands.

### 4.6. Clinical Implications

The results of this study provide relevant information for clinicians considering ZLS for posterior implant-supported single crowns. First, all groups exhibited fracture loads that exceeded the range of typical posterior masticatory forces in adults. This suggests that monolithic ZLS crowns, when fabricated with sufficient occlusal thickness and supported by titanium abutments, may offer a clinically acceptable level of mechanical performance for posterior implant restorations.

Second, the absence of significant differences between adhesive and self-adhesive cements indicates that, under standardized conditions, the strength of ZLS crowns may be less dependent on the type of resin cement than previously assumed. Clinically, this finding may broaden the range of viable cementation protocols for implant-supported ZLS crowns, although appropriate adhesive procedures remain advisable.

Third, the finding that mechanical aging did not significantly affect failure loads must be interpreted with caution but may suggest that ZLS monolithic crowns maintain stable behavior under moderate cyclic loading. This supports their potential use in patients without extreme occlusal demands.

However, these implications must be balanced with the limitations of the present study. Because loading was purely axial and thermocycling was not performed, the present results should not be directly extrapolated to patients with parafunction, pronounced occlusal wear, or non-axial loading patterns. Additionally, clinical scenarios often involve anatomical variability, antagonist diversity, and more complex loading conditions.

Despite these considerations, the present findings support the use of ZLS as a viable alternative for posterior implant-supported crowns when clinicians seek a restorative option that balances translucency with adequate mechanical properties, particularly in cases requiring enhanced esthetics without compromising structural performance.

### 4.7. Future Research Directions

The present study provides foundational data regarding the fracture resistance of monolithic ZLS implant-supported crowns; however, additional research is required to expand and validate these findings.

Future studies should incorporate loading protocols that simulate the complexity of oral function, including oblique and lateral forces, which are essential for understanding the true fatigue behavior of implant-supported ceramic restorations.

The effect of thermomechanical aging must also be addressed. Combined thermal cycling and cyclic loading would allow a more realistic evaluation of subcritical crack propagation, degradation of the resin–ceramic interface, and long-term stability in conditions that better mimic the oral environment.

Variations in crown geometry, occlusal anatomy, and cement space should be systematically explored. These parameters influence stress distribution and failure patterns and may interact with material properties in clinically significant ways.

Additional work is necessary to compare ZLS with other monolithic materials—such as lithium disilicate, zirconia-toughened lithium silicate, and translucent zirconia—under equivalent testing conditions. Such comparative data would help clinicians make evidence-based decisions regarding material selection for posterior implant-supported crowns.

Furthermore, different implant–abutment configurations, including hybrid abutments, customized zirconia abutments, and variable implant–abutment connection geometries, should be tested to determine whether the supporting structure amplifies or mitigates mechanical differences among materials.

Finally, well-designed prospective clinical trials with long-term follow-up are essential to validate the in vitro results. Clinical performance data on survival, chipping rates, antagonist wear, and complications are critical to establishing the long-term viability of ZLS as a posterior implant restorative material.

### 4.8. Novelty and Clinical Relevance

This study contributes new evidence on the mechanical behavior of monolithic ZLS crowns cemented onto titanium bases, a topic for which current scientific literature remains limited. Unlike previous investigations that often combine different abutment geometries, crown designs or mixed loading conditions, the present study employed a standardized posterior crown morphology and controlled axial load-to-fracture protocol, allowing the isolated assessment of the effect of cement type and cyclic mechanical aging. The use of two clinically relevant resin cements with distinct bonding mechanisms further enhances the applicability of the findings by reflecting common restorative strategies used in posterior implant-supported crowns. Clinically, the high fracture resistance values observed in all groups—consistently above reported maximal posterior biting forces—suggest that monolithic ZLS crowns may perform reliably under physiological occlusal loads when supported by titanium abutments. These findings support the indication of ZLS restorations in posterior regions for patients without severe parafunctional habits. However, given that the testing conditions involved purely axial forces and did not incorporate thermo-mechanical aging or oblique loading, the clinical relevance should be interpreted cautiously and validated through long-term in vivo studies.

## 5. Conclusions

Based on the findings of this in vitro study, the following conclusions can be drawn:

- Monolithic ZLS crowns cemented onto straight titanium abutments exhibited high fracture resistance under axial compressive loading, with all groups exceeding reported posterior occlusal forces.
- Neither the cement type (adhesive or self-adhesive) nor the applied cyclic mechanical aging protocol produced statistically significant differences in fracture resistance.
- All specimens failed through cohesive fracture of the ceramic material, indicating that the intrinsic properties of ZLS predominantly governed failure behavior.
- These findings apply only to the specific conditions tested—standardized crown geometry, straight abutments, purely axial loading, and absence of thermomechanical aging—and should not be generalized to other clinical scenarios.
- Further studies incorporating oblique loads, different crown designs, thermo-mechanical fatigue and in vivo validation are required to confirm the clinical applicability of these results.

## Figures and Tables

**Table 1:** Mean Fracture Resistance of ZLS Crowns According to Type of Cement Used.

Cement Type	*n*	Average Strength **	*p*-Value *
Calibra^®^ Ceram	20	1714.47 ± 169.65	0.102
Calibra^®^ Universal	20	1538.44 ± 140.10	

* T-Student test. ** Data are shown as mean ± SD.

**Table 2:** Mean Fracture Resistance of ZLS Crowns According to Application of Artificial Aging.

Aging Condition	*n*	Average Strength **	*p*-Value *
Aged	20	1649.53 ± 199.43	0.674
Not aged	20	1603.38 ± 109.37	

* T-Student test. ** Data are shown as mean ± SD.

**Table 3:** Average Fracture Resistance Combining Cement Type and Artificial Aging.

Group	Cement Type	Aging	*n*	Average Strength **	*p*-Value *
CCE	Calibra^®^ Ceram	Yes	10	1770.21 ± 352.58	0.431
CCNE	Calibra^®^ Ceram	No	10	1658.73 ± 118.79	
CUE	Calibra^®^ Universal	Yes	10	1528.86 ± 235.14	
CUNE	Calibra^®^ Universal	No	10	1548.03 ± 203.49	

* Kruskal–Wallis test. ** Data are shown as mean ± SD.

## Data Availability

The original contributions presented in this study are included in the article. Further inquiries can be directed to the corresponding author.

## Abbreviations

The following abbreviations are used in this manuscript:

ZLS	Zirconia-reinforced lithium silicate
CAD/CAM	Computer-Aided Design/Computer-Aided Manufacturing
SD	Standard Deviation
SPSS	Statistical Package for the Social Sciences
ADA	American Dental Association
ISO	International Organization for Standardization
MPa	Megapascal
N	Newton
Hz	Hertz
mm	Millimeter
GPa	Gigapascal
CE	Cemented with Calibra^®^ Ceram
CU	Cemented with Calibra^®^ Universal
CCE	Calibra^®^ Ceram, aged
CCNE	Calibra^®^ Ceram, non-aged
CUE	Calibra^®^ Universal, aged
CUNE	Calibra^®^ Universal, non-aged
USA	United States of America
UV	Ultraviolet
LED	Light-Emitting Diode

## References

[1] Sailer I., Makarov N.A., Thoma D.S., Zwahlen M., Pjetursson B.E. (2015). All-ceramic or metal-ceramic tooth-supported fixed dental prostheses (FDPs)? A systematic review of the survival and complication rates. Part I: Single crowns (SCs). Dent. Mater..

[2] Laumbacher H., Scholz K.J., Knüttel H., Rosentritt M. (2025). Clinical outcomes and complications of tooth- and implant-supported lithium (di)silicate based single crowns: An overview of systematic reviews. J. Dent..

[3] Sailer I., Philipp A., Zembic A., Pjetursson B.E., Hämmerle C.H.F., Zwahlen M. (2009). A systematic review of the performance of ceramic and metal implant abutments supporting fixed implant reconstructions. Clin. Oral Implant. Res..

[4] Zarone F., Ruggiero G., Leone R., Breschi L., Leuci S., Sorrentino R. (2021). Zirconia-reinforced lithium silicate (ZLS) mechanical and biological properties: A literature review. J. Dent..

[5] Zimmermann M., Koller C., Mehl A., Hickel R. (2017). Indirect zirconia-reinforced lithium silicate ceramic CAD/CAM restorations: Preliminary clinical results after 12 months. Quintessence Int..

[6] Manziuc M., Kui A., Chisnoiu A., Labuneț A., Negucioiu M., Ispas A., Buduru S. (2023). Zirconia-Reinforced Lithium Silicate Ceramic in Digital Dentistry: A Comprehensive Literature Review of Our Current Understanding. Medicina.

[7] Elsaka S.E., Elnaghy A.M. (2016). Mechanical properties of zirconia reinforced lithium silicate glass-ceramic. Dent. Mater..

[8] Preis V., Hahnel S., Behr M., Bein L., Rosentritt M. (2017). In-vitro fatigue and fracture testing of CAD/CAM-materials in implant-supported molar crowns. Dent. Mater..

[9] Gomes R.S., Souza C.M.C., Bergamo E.T.P., Bordin D., Del Bel Cury A.A. (2017). Misfit and fracture load of implant-supported monolithic crowns in zirconia-reinforced lithium silicate. J. Appl. Oral Sci..

[10] Deste Gökay G., Gökçimen G., Oyar P., Durkan R. (2024). Comparison of fatigue lifetime of new generation CAD/CAM crown materials on zirconia and titanium abutments in implant-supported crowns: A 3D finite element analysis. Biomed. Tech..

[11] (2007). Dentistry—Water-Based Cements.

[12] Rubió-Ferrer G., Rovira-Lastra B., Khoury-Ribas L., Flores-Orozco E.I., Ayuso-Montero R., Martinez-Gomis J. (2024). Reference values and reliability of occlusal force distribution and occlusal time measured by the T-Scan system in adults with healthy dentition. J. Prosthodont..

[13] Capobianco V., Baroudi K., Santos M.J.M.C., Rubo J.H., Rizkalla A.S., Dal Piva A.M.O., Vitti R.P., Tribst J.P.M., Santos G.C. (2023). Post-fatigue fracture load, stress concentration and mechanical properties of feldspathic, leucite- and lithium disilicate-reinforced glass ceramics. Heliyo.

[14] Sotto-Maior B.S., Carneiro R.C., Francischone C.E., Assis N.M.S.P., Devito K.L., Senna P.M. (2019). Fatigue Behavior of Different CAD/CAM Materials for Monolithic, Implant-Supported Molar Crowns. J. Prosthodont..

[15] Baroudi K., Almeida N.R., de Abreu L.S., Wandscher V.F., Ramos N.C., Padmanabhan V., Bucholz C.A., Amaral M. (2025). Clinical Adjustment of Zirconia-Reinforced Lithium Silicate and Lithium Disilicate Restorations Should Be Performed Before Crystallization. Materials.

[16] Rizzatto L.V., Meneghetti D., Di Domênico M., Facenda J.C., Weber K.R., Corazza P.H., Borba M. (2023). Effect of the type of resin cement on the fracture resistance of chairside CAD-CAM materials after aging. J. Adv. Prosthodont..

[17] Springall G.A.C., Yin L. (2018). Nano-scale mechanical behavior of pre-crystallized CAD/CAM zirconia-reinforced lithium silicate glass ceramic. J. Mech. Behav. Biomed. Mater..

[18] Bulut A.C., Atsü S.S. (2021). Occlusal Thickness and Cement-Type Effects on Fracture Resistance of Implant-Supported Posterior Monolithic Zirconia Crowns. Int. J. Oral Maxillofac. Implant..

[19] Bajraktarova-Valjakova E., Korunoska-Stevkovska V., Kapusevska B., Gigovski N., Bajraktarova-Misevska C., Grozdanov A. (2018). Contemporary Dental Ceramic Materials, A Review: Chemical Composition, Physical and Mechanical Properties, Indications for Use. Open Access Maced. J. Med Sci..

[20] Chen X.P., Xiang Z.X., Song X.F., Yin L. (2020). Machinability: Zirconia-reinforced lithium silicate glass ceramic versus lithium disilicate glass ceramic. J. Mech. Behav. Biomed. Mater..

[21] Rodríguez-Rojas F., Borrero-López O., Sánchez-González E., Hoffman M., Guiberteau F. (2022). On the durability of zirconia-reinforced lithium silicate and lithium disilicate dental ceramics under severe contact. Wear.

[22] Spitznagel F.A., Bonfante E.A., Vollmer F., Gierthmuehlen P.C. (2022). Failure Load of Monolithic Lithium Disilicate Implant-Supported Single Crowns Bonded to Ti-base Abutments versus to Customized Ceramic Abutments after Fatigue. J. Prosthodont..

[23] Leelaponglit S., Angkananuwat C., Krajangta N., Paaopanchon C., Ackapolpanich T., Champakerdsap C., Klaisiri A. (2024). Comparison of mechanical properties between zirconia-reinforced lithium silicate glass-ceramic and lithium disilicate glass-ceramic: A literature review. Oral Sci. Rep..

